# Statistical resolutions for large variabilities in hair mineral analysis

**DOI:** 10.1371/journal.pone.0208816

**Published:** 2018-12-26

**Authors:** Tsuyoshi Nakamura, Tomomi Yamada, Koshi Kataoka, Koichiro Sera, Todd Saunders, Toshihiro Takatsuji, Toshio Makie, Yoshiaki Nose

**Affiliations:** 1 Faculty of Environment Science, Nagasaki University, Nagasaki, Japan; 2 Department of Medical Innovation, Osaka University Hospital, Osaka, Japan; 3 School of Medicine, Yokohama City University, Yokohama, Japan; 4 Cyclotron Research Center, Iwate Medical University, Iwate, Japan; 5 School of Medicine, Nagasaki University, Nagasaki, Japan; 6 Suzuka National Hospital, Suzuka, Mie, Japan; 7 School of Medicine, Kyushu University, Fukuoka, Japan; University of Pisa, ITALY

## Abstract

Measuring biomaterials is usually subject to error. Measurement errors are classified into either random errors or biases. Random errors can be well controlled using appropriate statistical methods. But, biases due to unknown, unobserved, or temporary causes, may lead to biased conclusions. This study describes a verification method to examine whether measurement errors are random or not and to determine efficient statistical methods.

A number of studies have dealt with associations between hair minerals and exposures such as health, dietary or environmental conditions. Most review papers, however, emphasize the necessity for validation of hair mineral measurements, since large variations can cause highly variable results. To address these issues, we answer the following questions:

1. How can we ascertain the reliability of measurements?
2. How can we assess and control the variability of measurements?
3. How do we efficiently determine associations between hair minerals and exposures?
4. How can we concisely present the reference values?

Since hair minerals all have distinctive natures, it would be unproductive to examine each mineral individually to find significant and consistent answers that apply to all minerals. To surmount this difficulty, we used one simple model for all minerals to explore quantitative answers. Hair mineral measurements of six-year-old children were analyzed based on the statistical model. The analysis verified that most of the measurements were reliable, and their inter-individual variations followed two-parameter distributions. These results allow for sophisticated study designs and efficient statistical methods to examine the effects of various kinds of exposures on hair minerals.

## Introduction

Kempson et al. [1] remark “*Hair analysis has received a large amount of academic and commercial interest for wide-ranging applications*, *however the degree of success of analytical interpretation with hair mineral analysis has been quite minimal with respect to extent of such endeavors*”. They attribute this limited success to large variabilities in hair mineral measurements and are more concerned with the biochemical aspects of hair mineral analysis to reduce and regulate the variations. Our primary concern in this study is to examine variations in hair mineral measurements from a statistical perspective. Since scientific research deduces based on measurements, the credibility of the research results depends on the reliability of the measurements. And since most measurements are subject to errors, researchers need to draw conclusions taking into account the effects of these measurement errors. In biomedical research, individual differences can increase the complexity as well as the magnitude of the errors. Individual differences sometimes follow natural laws and result in a simple statistical distribution, hereafter referred to as the *inter-individual* distribution. Since measurement errors and inter-individual distributions are the major sources of hair mineral measurement variability, it is necessary to fully understand these variabilities in order to perform valid and efficient statistical analyses of hair mineral measurements, however clarifying the nature of measurement errors and inter-individual distribution for over 30 different hair minerals is not straightforward. Consequently, nonparametric methods are generally used in statistical analysis of hair mineral measurements, since they are valid regardless of the nature of the variability. However, nonparametric methods are not very efficient for detecting the effects of exposures such as therapeutic effects, dietary intakes or environmental changes on study subjects. This disadvantage seems to be one of the main causes that led Kempson et al. [1] to remark about minimal success.

Previously we conducted a cohort study of 834-mother-infant pairs to determine the association between hair minerals at one-month and the onset of atopic dermatitis at ten-months after birth with hair minerals measured using the Particle Induced X-ray Emission (PIXE) method [2]. In executing the study, we encountered large variations in hair mineral measurements as repeatedly pointed out in the review literature [1, 3–5]. This experience prompted us to examine the statistical nature of the variations in hair mineral measurements and develop statistical methods to obtain results corrected for the biases caused by these variations [6–9]. In this study, we are concerned with four questions:

1. How can we ascertain the reliability of hair mineral measurements?
2. How can we assess and control the variability of hair mineral measurements?
3. How do we efficiently determine associations between hair minerals and exposures?
4. How can we concisely present the reference values, or the coverage intervals?

A number of studies have been devoted to questions 3 and 4. Some describe gender-specific reference values [10,11,12,13,14,15,16,17,18,19], and some extensively examined the effects of dietary intake on hair mineral concentrations [10, 11, 20, 21, 22, 23]. Others explored the effects of environmental factors on hair minerals [11, 16, 24, 25, 26]. The most common subjects were associations between hair minerals and health conditions [14, 22, 24, 27, 28, 29, 30, 31, 32]. Commonalities among most of those studies were the use of nonparametric methods for statistical analysis without clarifying the nature of the variability in hair mineral measurements. Unfortunately there are few studies describing the application of statistical methods to answer Q1 and Q2.

To address these four questions, this paper proposed using a simple statistical model to examine the data. The results revealed that most intra-individual variations in hair mineral measurements were normal random errors. Furthermore, the inter-individual variation was approximated by an ordinary two-parameter distribution for each mineral. These results make it possible to apply efficient statistical methods, rather than nonparametric techniques, for the analysis of hair mineral measurements.

Traditionally, Inductively Coupled Plasma Mass Spectrometry (ICP-MS) and Inductively Coupled Plasma Atomic Emission Spectrometry (ICP-AES) have been the multi-element instruments most frequently used for hair mineral analysis, and most of the studies targeted by the above review literature [1, 3–5] used ICP-MS or ICP-AES. Although this study deals with hair mineral measurements obtained using PIXE, the statistical methods described in this study can be immediately applied without modification to measurements obtained using either ICP-MS or ICP-AES.

## Methods

### Subjects and PIXE analysis

The original cohort sample consisted of 834 mother-infant pairs, who were recruited by their obstetricians at the infants’ first one-month national health checkups starting in November 2005, and who participated in their ten-month national follow up health checkups [2]. The subjects lived in Fukuoka city and voluntarily presented their hair samples at both checkups, which were performed by 13 obstetricians and 77 pediatricians at 90 hospitals throughout Fukuoka. Fukuoka is located on Kyushu Island, faces the Sea of Japan, and has a population of about 2 million. It was elected as the best city to live in Asia several times, because of the moderate climate, good infrastructure and non-polluting industry.

In 2011, six years after the initial research, we sampled 209 then 6-year-old children from the original cohort using PIXE. Hair samples were collected from the base of the scalp close to the occipital region at a length not exceeding 5 cm using a pair of stainless steel scissors. For target preparation, the root-sides of the hairs were attached to the bottom of a holder with adhesive tape and then fixed on the topside in order to avoid overlapping. The target samples were analyzed by the standard-free PIXE method described in S2 Text, in the Nishina Memorial Cyclotron Center, Iwate Medical University, Japan.

Each of the 209 children’s hair strand samples was divided into two specimens for PIXE analysis to obtain two independent measurements for each subject. We statistically analyzed the measurements based on a simple statistical model described S1 Text.

### Detectability

One issue particular to statistical analysis of hair minerals is how to treat “*0*”, since *0* does not necessarily mean exactly *0*, rather it indicates a small amount less than the detection limit. When the two measurements of a pair are both *0*, we call the pair *Zero*. Since the difference between two *0*’s is exactly *0*, it seems operationally relevant to consider their true value to be also *0* and disregard *0*’s in an analysis of differences between pair of measurements to avoid analyzing variations in measurements below the detection limit. Treatment of *Zero* in applications is addressed in the Discussion.

### Validity

First we examined the differences between the two measurements of each pair; see S1 Text for the statistical model and calculations. When the differences were approximately normally distributed for a mineral, the mineral was termed *valid*, since the differences were considered to be caused solely by chance. The Shapiro-Wilk W test was used for testing the normality [33]. Pairs where the differences between the two measurements were too large to fit the normal distribution were termed *outliers* and excluded in the testing for normality. Pairs other than outliers were termed *valid*. However, since outliers sometimes carry the most significant information [34], they should not be simply disregarded. Treatment of the outliers in applications is addressed in the Discussion.

### Tractability

Only valid pairs were treated in the following statistical analysis. For a valid pair, the mean of the two measurements is more reliable than the individual measurements. Thus, we obtained a histogram of the mean of the two measurements of each pair and determined a parametric distribution that well fit it. Since extreme values are inevitable for some minerals due to unusual food consumption, medication, hair treatments, environment, and other exposures [1], a few extremely large values were disregarded in the fitting analysis. Kolmogorov D test and Cramer-von Mises W test were used for testing the fitness of the log-normal and Weibull distribution, respectively [33]. When a parametric distribution is well fitted for a mineral, we call the mineral statistically *tractable*. S1 Fig illustrates the flow of the statistical analysis.

This study was approved by the Institutional Review Board of Kyushu University and the informed consent was provided on a written document and signed by each mother.

## Results

S2 Fig presents a scatter plot for each pair of measurements for each mineral with units in *ppm*. The number of pairs used in each plot is that of positive pairs described in Table 1. Each mineral shows a peculiar variation between the two measurements obtained from the same subject at the same time. S2 Fig clearly shows how large the differences may be between two measurements of the same subject. Using Mg as an example, a subject’s first measurement could be 40 ppm while the second could be less than 5 or larger than 100. Uncertainties in measurements are unavoidable unless their major causes are identified and corrected. But this is often difficult even in physical sciences, since, as Bailey [34] points out, outliers may also be a sign of healthy science.

**Table 1:** The number of subjects and the transformations used in S3 and S4 Figs.

	Detectability		Validity			Tractability			
	Positive pairs	%	Trans form.	Valid pairs	Excluded %	Transform.	Distribution	Included pairs	Excluded %
**Na**	209	100%		206	1.4%		Lognormal	188	8.7%
**Mg**	188	90%		186	1.1%		Weibull	186	0.0%
**Al**	161	77%		161	0.0%		Weibull	161	0.0%
**Si**	209	100%		208	0.5%		Lognormal	205	0.5%
**P**	143	68%		141	1.4%		Weibull	141	0.0%
**S**	209	100%		209	0.0%		Normal	209	0.0%
**Cl**	209	100%		193	7.7%		Lognormal	193	0.0%
**K**	209	100%	log	202	3.3%		Lognormal	180	11.0%
**Ca**	209	100%		200	4.3%		Lognormal	200	0.0%
**Ti**	209	100%	log	203	2.9%		Normal	182	10.3%
**V**	144	69%		144	0.0%		Weibull	144	0.0%
**Cr**	203	97%		202	0.5%	√	Normal	202	0.0%
**Mn**	81	39%		79	2.5%		Weibull	79	0.0%
**Fe**	209	100%		198	5.3%		Lognormal	198	0.0%
**Co**	150	72%		150	0.0%	√	Weibull	150	0.0%
**Ni**	202	97%		199	1.5%		Weibull	196	1.5%
**Cu**	209	100%	log	206	1.4%		Lognormal	186	9.7%
**Zn**	209	100%		203	2.9%		Normal	198	2.5%
**Ga**	185	89%		184	0.5%		Weibull	184	0.0%
**As**	145	69%		143	1.4%		Weibull	143	0.0%
**Se**	205	98%		205	0.0%		Weibull	205	0.0%
**Br**	209	100%		197	5.7%		Lognormal	193	0.0%
**Rb**	179	86%		170	5.0%		Weibull	170	0.0%
**Sr**	208	100%		203	2.4%	√	Weibull	203	0.0%
**Nb**	144	69%		140	2.8%		Weibull	140	0.0%
**Mo**	171	82%		168	1.8%		Weibull	168	0.0%
**Hg**	205	98%		205	0.0%		Weibull	205	0.0%
**Pb**	209	100%		205	1.9%		Weibull	202	1.5%

To gain better insight into the significance of these variations, we classified the variations into three categories. The “*Large variation*” category consists of Na, Mg, S, Ti, and Pb. The “*Linear regression*” category consists of Si, Cl, K, Ca, Fe, Cu, Zn, Br and Sr. And the “*Regression to the mean*” category consists of Al, P, V, Cr, Mn, Co, Ni, Ga, As, Se, Rb, Mo and Hg. Mineral measurements in the *Large variation* category are not very reliable because of the large variations. Measurements in the *Linear regression* category appear more reliable and suitable for linear regression analysis. However, for some minerals, this good appearance is due to the presence of very large measurements which obscure the relatively large variations of smaller measurements. For instance, scatter plots for Si<300, Cl<1500, K<300, Fe<20, Br<15 and Sr<10, are all close to those in the first category.

The *Regression-to-the mean* phenomenon of the minerals in the last category is rarely described in applications. For minerals in this category, variations may be so large that one measurement of a pair might be 0, while the other may be 0 or the nearly maximum of the mineral.

These results suggest that hair mineral measurements are subject to large variations and the statistical nature of the variations also vary among minerals.

### Detectability

Table 1 summarizes the results. Among the 32 minerals, Ag, Cd, I and Ba were not detected in any pairs and excluded. Table 1 column 2 shows the number of positive pairs, and column 3 the proportion of them. 17 minerals (60%) and 27 minerals (96%) have a positive rate greater than or equal to 97% and 68%, respectively.

### Validity

S3 Fig shows the histogram of the differences, overwritten with a normal distribution, for each mineral that is associated with mean, *SD*, sample size, skewness, kurtosis, minimum, maximum and *p*-value by the Shapiro-Wilk W test of normality. A two-tailed p<0.05 is considered significant, and all minerals were confirmed to be valid. No pair was excluded for the 6 minerals, less than 2% were excluded for 17 minerals and less than or equal to 5% were excluded for 25 minerals. As described in S1 Text, *SD*^2^/2 is an unbiased estimate of the intra-individual variance σ^2^, or the variance due to locations within a subject. The 4th column of Table 1 indicates that three minerals, Ca, Ti and Cu, were log transformed to confirm the validity. The 5th and 6th columns indicate the number of valid pairs and the proportion of the excluded pairs, or outliers, in the positive pairs.

### Tractability

S4 Fig presents the histogram of the mean of the two measurements of a pair, overwritten with a parametric distribution for each mineral. Associated with them are mean, median, *SD*, minimum, maximum, type of distribution, parameter values, *p*-value obtained from the statistical test for the fitness. The fitness for the Normal, Weibull, and Lognormal distributions were tested by Kolmogorov D, Cramer-von Mises W and Kolmogorov D tests, respectively. The *SD* will be denoted by *SD*_*B*_ to distinguish it from the *SD* shown in S3 Fig. The 7^th^ column shows that Cr, Co and Sr were square-root transformed to analyze the tractability. The 8^th^ column shows the type of distribution fitted for the mineral. The 9^th^ column shows the number of pairs used to confirm the tractability and the 10^th^ the proportion of excluded pairs among the valid pairs. No pair was excluded for 20 minerals, less than or equal to 2.5% were excluded for 24 minerals and less than 10% were excluded for 26 minerals. All excluded pairs were too large to fit the distribution, and all minerals were confirmed to be tractable. Distribution types used for the fitting are Normal, Log-normal, Weibull, √-Normal or √-Weibull. As described in S1 Text, the inter-individual variance *σ_B_*^2^, the variance due to individual differences, is obtained by *SD*_*B*_^2^—*SD*^2^/2.

The results described in this section apply to positive measurements. In other words, comparing positive measurements among populations is performed using the two parameters of those distributions. On the other hand, for *0*, the proportion of *0*’s is compared among populations.

## Discussion

We described a verification method to examine the validity of measurements. The method is applicable to ICP-MS, ICP-AES, or any other methods. To demonstrate how to use this method, it was applied to hair mineral measurements obtained using PIXE. This verification method can be used to assess the validity of data as well as to determine the most efficient statistical method so that biomedical professions may correctly and adequately interpret the data.

### Detection rate and LOD

Table 1 reveals that some elements have low detection rates of positive pairs such as Mg, Al, P, V. Further studies should investigate the reason for these low rates from a biomedical or physical point of view to determine whether these elements should or should not be used for main analysis.

Measurements below LOD are frequently observed with hair element analysis. The appropriate treatment of them depends on the objective of the study. According to Molina-Villalba et al. [12], values below the LOD were *“assigned the LOD divided by the square root of 2*“. However, Varrica et al. [16] and Dongarrà et al. [18] set “*values below the detection limit… at one-third the detection level and treated (them) as real values*”.

In our study, we obtained hair strands from each subject and separated them to make two analytes from different locations. The difference in the true value between the two locations is the intra-individual variation that is estimated using the difference between measurements obtained from the analytes. We assigned measurements <LOD “0”. The results of this study suggested that the differences between observed values in the two locations were regarded as random errors, implying that the magnitude of the difference between the true value and “0” was relatively negligible as compared to that of the difference in observed values between the two locations.

### The four questions

To answer the first and the second questions described in the Introduction, we sampled two hair-strand specimens from each subject to obtain two statistically independent hair mineral measurements. If the distribution of the differences between the two measurements is approximately normally distributed then the differences are regarded as normal random errors; the measurements are considered valid and follow a simple random model described in S1 Text. These measurements are qualified for statistical analysis assuming this model.

A valid mineral is termed tractable when the distribution of the mean of the two measurements, after excluding the outliers, is well approximated by a parametric distribution. In this study, all minerals were confirmed tractable. That is, all of the minerals are approximated by either Normal, Lognormal or Weibull distribution, and it will be quite useful in applications that each distribution is determined by only two parameters. For instance, if exposures such as medical treatments, environmental changes, or dietary intakes affect some hair minerals, then the effects of the exposure can be assessed by comparing the two parameters of the distribution with those of the distribution without exposure. This answers the third question. It is also straightforward to calculate any coverage interval and percentile points from a parametric distribution. Coverage intervals adjusting for those exposures may also be obtained by modifying parameter values. This answers the fourth question.

### Disregarded measurements

The *0*’s are not used in fitting a parametric distribution, since interpretation regarding the biological abnormality of *0* depends on the objective of the study. For an essential mineral in clinical medicine, *0* should be regarded as abnormally low, whilst for a toxic element in an environmental science, *0* should be considered normal. In certain applications, it may be useful to consider jointly the number of *0*’s and the parameter values of a distribution fitted for the positive pairs.

When the differences between the two measurements are normally distributed, the pairs are termed valid. Conversely, when a difference is too large to fit the normal distribution, a detailed examination might find particular factors causing the unusually large differences between the measurements in the same subject. They could be experimental errors or unexpected responses.

In fitting a parametric distribution, too large measurements were excluded as outliers. However, those measurements may carry unobserved but significant information [34], unless they are caused simply by mistakes. Appropriate treatment of those measurements should be considered according to the objective of the study. Conversely, determining the distribution that most subjects follow makes it possible to identify outliers.

### Reliability of hair mineral analysis

When examining the reliability of hair mineral results from laboratories, a standard procedure is to split the hair sample of a single healthy volunteer, send them to different laboratories, compare the reported results, and conclude on the reliability of the hair mineral analysis of the laboratories [35–37]. This design of reliability analysis treats hair like blood. Most minerals are uniformly distributed in the blood in the same density regardless of volume. However, most minerals are not uniformly distributed in hair strands and the density may vary considerably between hair strands within a subject. Thus, to comprehensively understand the variations in hair mineral measurements, appropriate statistical analysis taking into account intra-individual variations is necessary. However, many studies rarely considered this aspect. They ascribe the differences in measurements between laboratories to systematic factors such as variations in sample preparations or calibration standards between laboratories.

We demonstrated a statistical analysis assuming a simple statistical model to assess the reliability of hair mineral measurements. The model requires two measurements from each subject. The results of this analysis verified that the differences are regarded as normal random errors. In other words, the hair mineral measurements obtained from PIXE analysis in this study are reliable. Therefore, results obtained from applying an appropriate statistical model to the measurements are also reliable.

### Large variations

Large intra-individual variations in hair minerals cause troubles in various applications. Dulgaszek et al. [5] concluded “Taking into account the high variability of results, further research on the content of elements in human hair should be continued”. Kempson et al. [1] overview possible causes of the variability of hair minerals, and suggest much more normalization and accurate quality controls for hair mineral analysis. Wołowiec et al. [4] review associations between the mineral composition of hair, and physical or mental disorders, and insist on the standardization of sample preparation. Mikulewicz et al. [38] review reference values of elements in human hair. They screened 52 studies to leave only five as eligible for their review. However, since the reported reference values still varied, they concluded it is necessary to further elaborate the standard procedures to validate hair mineral analysis.

To surmount these large intra-individual variations, it is crucial to understand the statistical nature of the variations in hair mineral measurements. We must first define the *true value* of hair mineral measurements for a subject, and then define the intra-individual and inter-individual variations. Statistical analysis using a simple random model (S1 Text) revealed that the intra-individual variations are mostly random, rather than caused by any temporal accident or systematic biases. More interestingly, the inter-individual variations are approximated by ordinary two-parameter distributions. In other words, those variations are statistically tractable, or controllable. The ratio of the inter-individual variance to the intra-individual variance will be particularly useful for designing studies taking into account the variability of hair mineral measurements.

### Hair minerals as possible biomarkers for intractable diseases

Intractable diseases refer mainly to rare diseases with unidentified preventions or treatments. Early and accurate diagnosis is critically important for those patients; however, the current situation for specific rare disease identification is not optimistic [39]. Tang and Makuuti [39] suggest that biomedical research on rare diseases will provide insights into underlying mechanisms, which may ultimately reveal possible avenues to therapeutics. Since a cohort study with the endpoint being the onset of a rare disease is difficult to perform, a case/control study is the only possible study design for finding risk factors for those diseases. The most difficult part in implementing the case/control study is the need for accurate information on the subject's environment or health condition a few months to a few years before the onset. In that respect, hair minerals are ideal biomarkers for case/control studies, since hair strands are usually easy to obtain, and hair minerals are measured using PIXE or ICP methods. Thus, it is expected that hair minerals will contribute to providing useful information for studies on underlying mechanisms of rare diseases, if a standard method for the analysis of hair mineral measurements is established.

If a study is designed to confirm hair minerals to be significant biomarkers for intractable diseases, then information on the nature of measurement errors and inter-individual distributions of hair minerals would be of critical importance [40, 41].

Dataset used in this study is presented in S1, S2 and S3 Dataset.

## Supporting information

S1 Text Statistical model. (DOCX)

S2 Text Standard-free PIXE method. (DOCX)

S1 Fig Flow of the statistical analysis. (TIF)

S2 Fig Scatter Plot between the two measurements of a pair. (TIF)

S3 Fig Histogram of the differences overwritten with a normal distribution for each mineral with descriptive statistics and *p*-value by the Shapiro-Wilk W test of normality. (TIF)

S4 Fig Histogram of the mean of the measurements and a fitted parametric distribution for each mineral. Distribution type, parameter values, descriptive statistics, statistical test for the fitness, and p-value obtained from the test are described.
(TIF)

S1 Dataset Original hair mineral data. (XLSX)

S2 Dataset Differences. (XLSX)

S3 Dataset Averages. (XLSX)
