# Supplementary material for: Statistical resolutions for large variabilities in hair mineral analysis
Source: PLoS One. 2018 Dec 26;13(12):e0208816. doi: 10.1371/journal.pone.0208816 (PMC6306225; doi:10.1371/journal.pone.0208816)
Supplement: S1 Text — (DOCX) [file pone.0208816.s001.docx]

**S1 Statistical Model**

Our statistical model is as follows:

Let $X_{ij}$denote a measurement of hair mineral for $j^{th}$ specimen (*j*=1, 2) of $i^{th}$ subject (*i=*1,…,n). We assume a simple model:

$X_{ij}=\mu_{i}+\varepsilon_{ij}$ (1)

Where$\mu_{i}$, termed *true value*, denotes the average of the mineral amount over all locations in the hairs of $i^{th}$ subject. $\varepsilon_{ij}$denotes a deviation from $\mu_{i}$.

It is assumed that $\varepsilon_{ij}$’s are independently and normally distributed as $\varepsilon_{ij}\sim N\left( 0, \sigma^{2} \right)$ and $\mu_{i}$ and $\varepsilon_{ij}$ are independent with each other. $\sigma^{2}$ is called the *intra-individual, or within-individual, variance* common to the subjects. Whilst, $Var\left( \mu_{i} \right)=\sigma_{B}^{2}$is called the *inter-individual, or the between-individual, variance.* It follows from the assumptions that the difference ${D_{i}=X}_{i1}-X_{i2}= \varepsilon_{i1}-\varepsilon_{i2}$ is also normally distributed as $N\left( 0, 2\sigma^{2} \right)$. It holds

$\sigma^{2}=Var(D_{i})/2$ (2)

Put $Y_{i}=(X_{i1}+X_{i2})/2$ and $\varepsilon_{i}=(\varepsilon_{i1}+\varepsilon_{i2})/2$. Since $\mu_{i}$ and $\varepsilon_{i}$ are independent, it follows

$\mathrm{Var}\left( Y_{i} \right)=Var\left( \mu_{i}+\varepsilon_{i} \right)=Var\left( \mu_{i})+Var(\varepsilon_{i} \right)=\sigma_{B}^{2}+\sigma^{2}/2$

Therefore

$\sigma_{B}^{2}=Var\left( Y_{i} \right)-\sigma^{2}/2$ (3)

Using (A2) and (A3), we obtain the intra- and inter- individual variances.

Suppose we compare a hair mineral between treated and control groups. Let *n* be the number of subjects for each group and *m* be the number of hair-strand analytes from each subject. Then the variance of the average of *nm* measurements is

V=__

for each group. If an expected difference between the groups is Δ, then *T*=Δ/$\sqrt{2V}$ should be large enough for the difference to be statistically significant. Sample size of the study is determined by considering the cost of increasing *n* and *m*.
