# Supplementary material for: Statistical resolutions for large variabilities in hair mineral analysis
Source: PLoS One. 2018 Dec 26;13(12):e0208816. doi: 10.1371/journal.pone.0208816 (PMC6306225; doi:10.1371/journal.pone.0208816)
Supplement: S2 Text — (DOCX) [file pone.0208816.s002.docx]

**S 2 Standard-free PIXE Method**

PIXE was applied for the analysis of elemental concentrations. Quantitative analyses of untreated hairs were performed on the basis of the standard-free method developed by Sera et al. [1**-**3]. Hairs were wiped well with acetone. For convenience and efficiency only 5–6 hairs were taped to a target holder. They were directly irradiated with proton beams, and emitted X-rays were measured with two Si(Li) detectors simultaneously. Zn was designated as an index element since it exists in all hair samples at levels over 50 ppm. Its concentration was first derived on the basis of the standard-free method [1], and those of all the other elements were derived using Zn as an internal standard^43^. The standard-free method makes use of the yields of continuous X-rays. The total yield in a spectrum is obtained by subtracting all X-ray peaks from the raw spectrum. The ratio of each peak yield to the total yield is used for the derivation of absolute values of concentration.

The yields of continuous X-rays are proportional to the number of electrons in the sample to the first approximation. In order to avoid ambiguities coming from absorption of X-rays and for accuracy of peak separation, a 300-μm thick Mylar film absorber is employed and the total yield of continuous X-rays of higher than 3 keV, where their spectral shape can be observed clearly, is used for quantitative analysis. In order to obtain the conversion coefficient for hair samples, many hair samples were analyzed by this method and the internal standard method which involves the preparation of the hair samples by chemical washing. The results obtained were comparable with each other including light elements [2].

Testing was performed using the facilities of the Nishina Memorial Cyclotron Center, Iwate, Japan. A small cyclotron provided a 2.9 MeV proton beam on a target after passing through a beam collimator of graphite. X-rays of energies higher than those of K−Kα were detected by a Si(Li) detector (0.0254 mm Be window) with 300-µm thick Mylar absorber. For lower-energy X-rays, a Si(Li) (0.008 mm Be) was used without an absorber, simultaneously.

SAPIX computer code was used for the spectrum analysis developed by SERA et al [2]. The performance of the code was evaluated by Blaauw et al. [4] Experimental errors are mainly from the spectrum fitting and the detection efficiencies. A detailed discussion on experimental accuracy in hair analysis are given in Sera et al. [1], and preparation for bio-samples and long hair are described in Sera et al. [5] and Sera et al. [6] respectively.

**References**

1. Sera K, Futatsugawa S, Matsuda K. Quantitative analysis of untreated bio-samples. Nucl. Instr. Meth. Phys. Res. 1999; B150: 226-233.

2. Sera K, Futatsugawa S. Personal computer aided data handling and analysis for PIXE. Nucl. Instr. Meth. Phys. Res. 1996; B109/110: 99-104.

3. Sera K, Futatsugawa S, Murao S. Quantitative analysis of untreated hair samples for monitoring human exposure to heavy metals. Nucl. Instr. Meth. Phys. Res. 2002: B189 174-170

4. Blaauw M, Campbell JL, Fazinić S, Jakšić M, Orlic I, Van Espen P. The 2000 IAEA intercomparison of PIXE spectrum analysis software. Nucl Instrum Methods Phys Res. 2002; B189: 113-22.

5. Sera K, Futatsugawa S, Matsuda K, Miura Y. Standard-free Method of Quantitative Analysis for Bio-samples. Int'l Journal of PIXE 1996; 6: 467-481.

6. Sera K, Terasaki K, Sasaki T, Goto S, Saitoh Y, Itoh J. Studies on Changes of Elemental Concentration in a Human Body by Means of Analyses of Long Hairs on the Basis of the Standard-Free Method. Int'l Journal of PIXE 2009; 19: 17-27.
