# Supplementary figures and images for: Statistical resolutions for large variabilities in hair mineral analysis
Source: PLoS One. 2018 Dec 26;13(12):e0208816. doi: 10.1371/journal.pone.0208816 (PMC6306225; doi:10.1371/journal.pone.0208816)

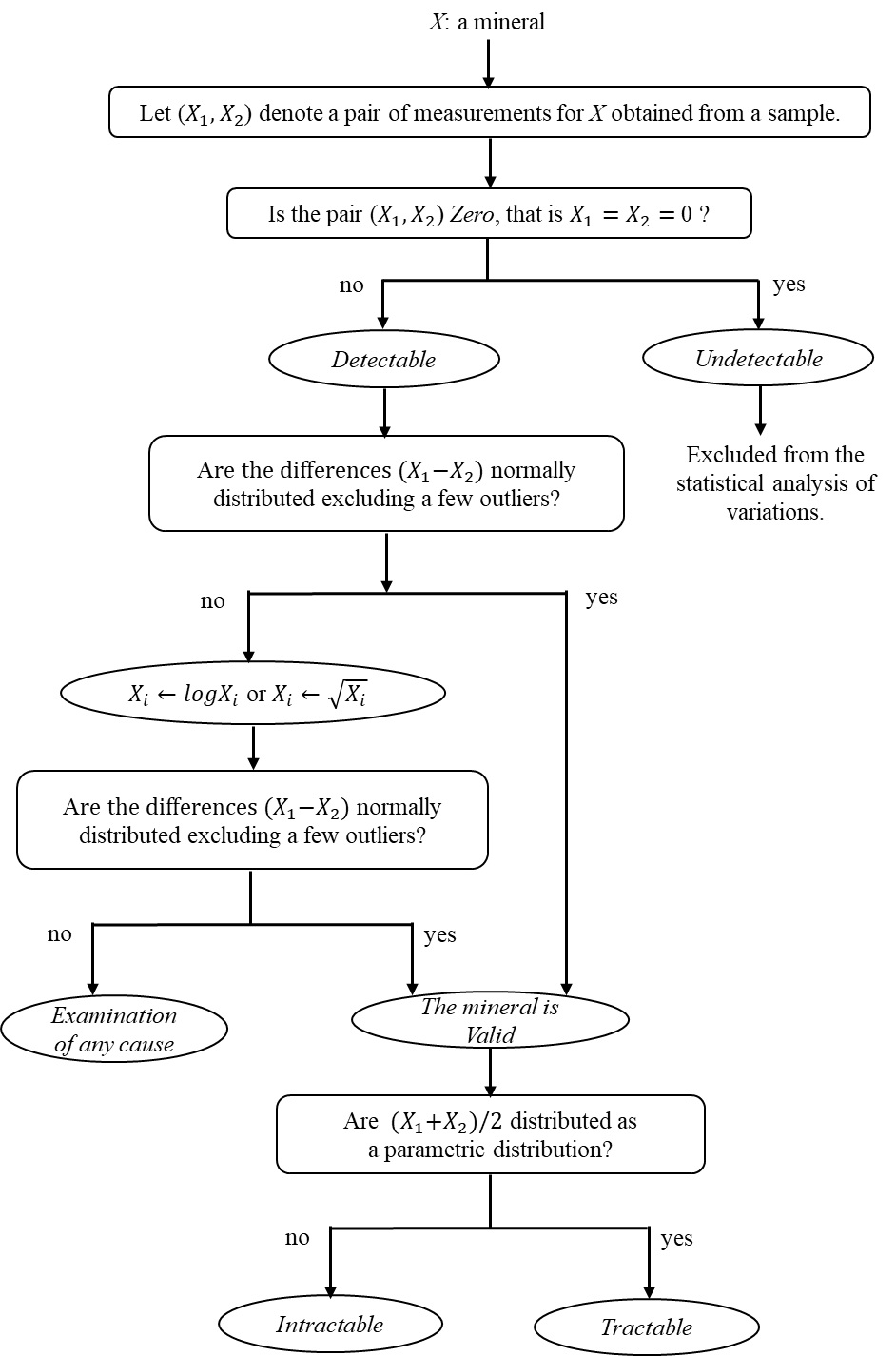

Supplement: S1 Fig — (TIF) [file pone.0208816.s003.tif]

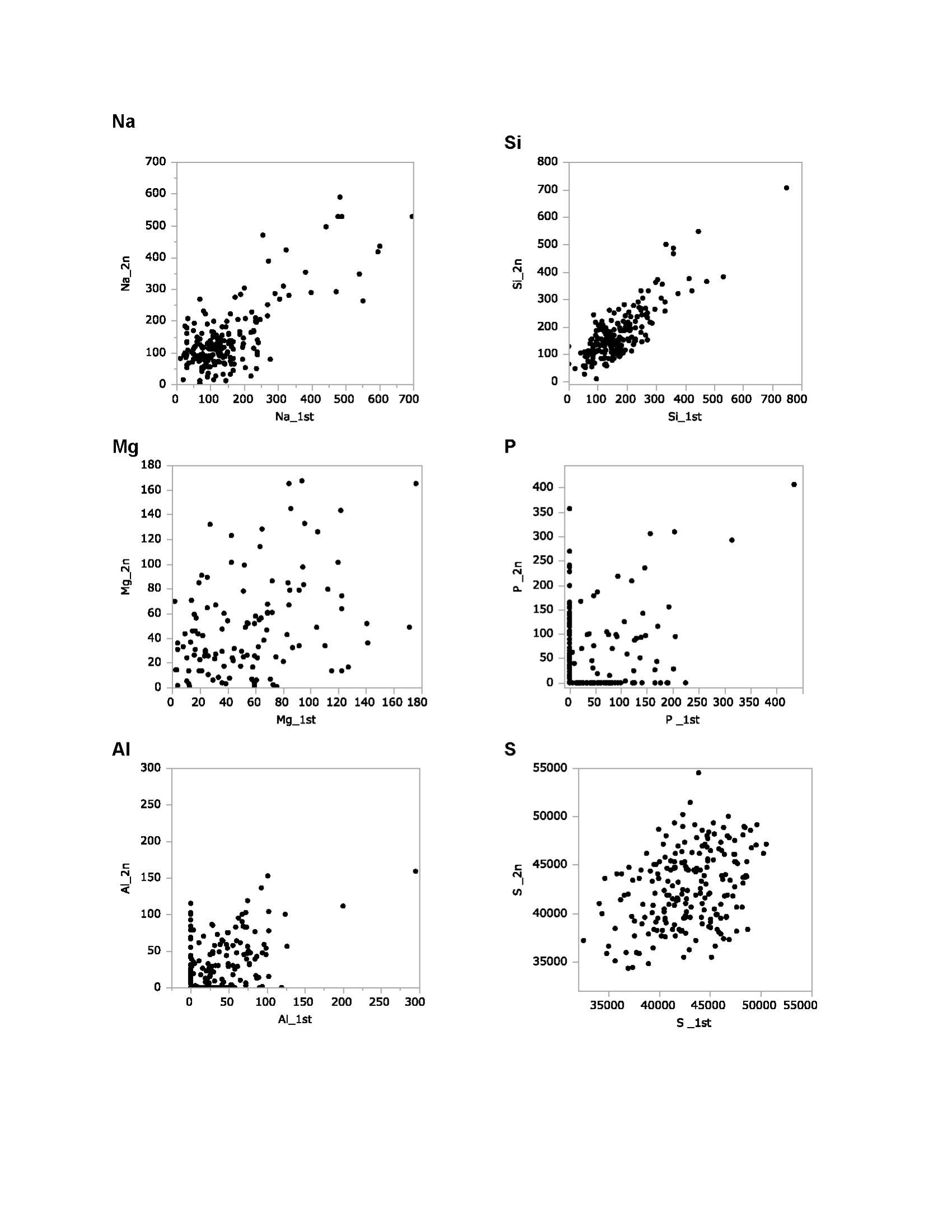

Supplement: S2 Fig — (TIF) [file pone.0208816.s004.tif]

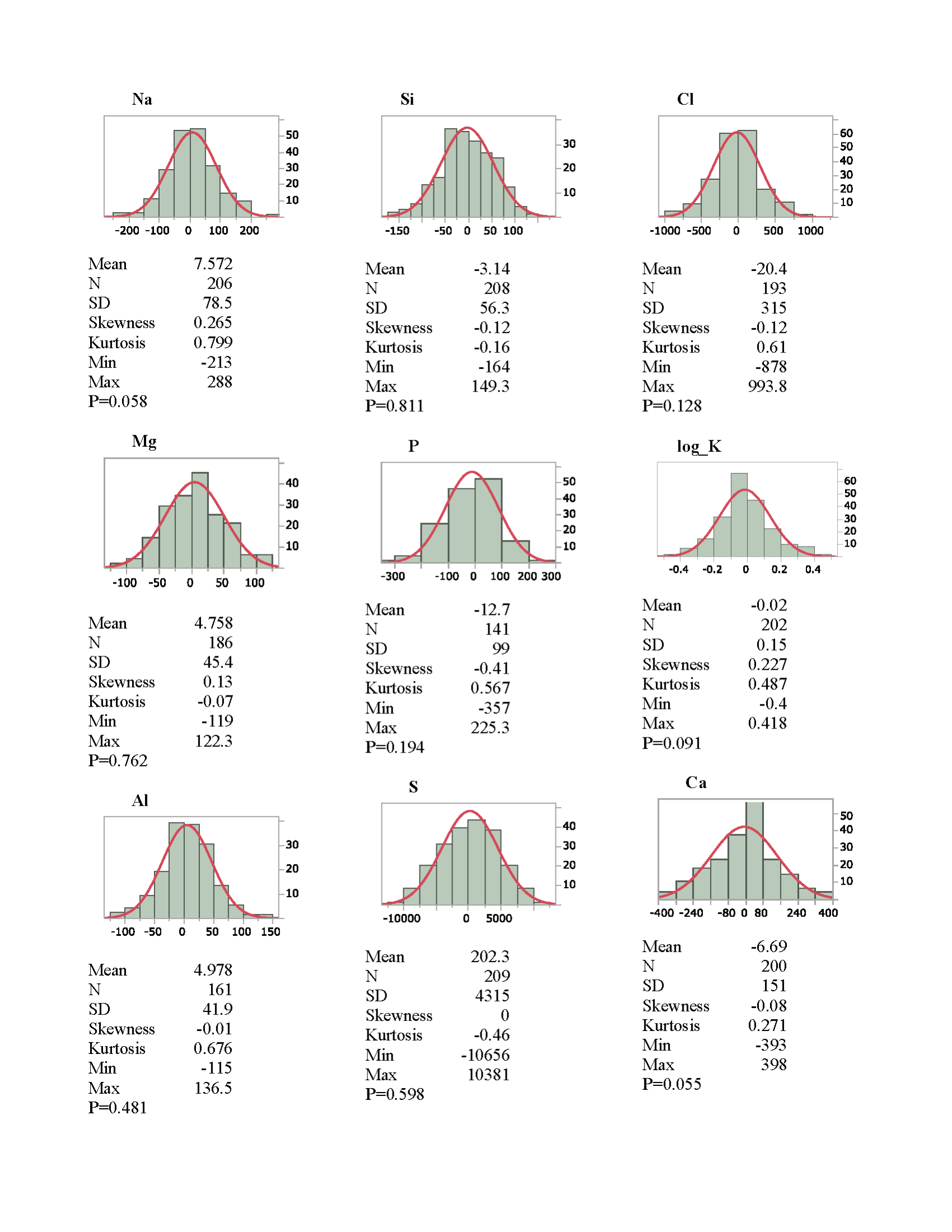

Supplement: S3 Fig — (TIF) [file pone.0208816.s005.tif]

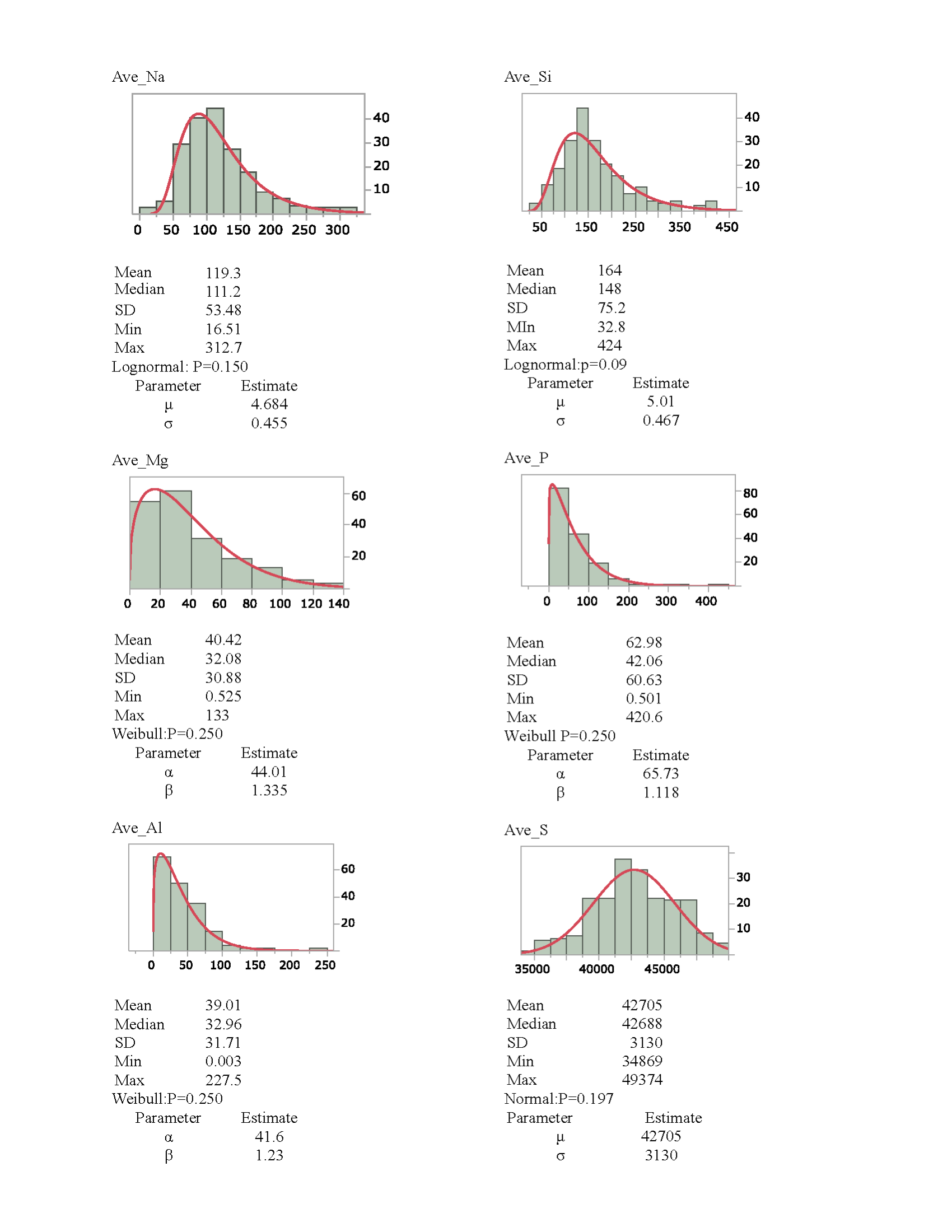

Supplement: S4 Fig — Distribution type, parameter values, descriptive statistics, statistical test for the fitness, and p-value obtained from the test are described. (TIF) [file pone.0208816.s006.tif]
